# Evaluation of three molecular markers for identification of European primary parasitoids of cereal aphids and their hyperparasitoids

**DOI:** 10.1371/journal.pone.0177376

**Published:** 2017-05-31

**Authors:** Zhengpei Ye, Ines M. G. Vollhardt, Zeljko Tomanovic, Michael Traugott

**Affiliations:** 1 Mountain Agriculture Research Unit, Institute of Ecology, University of Innsbruck, Innsbruck, Austria; 2 Agroecology, Department of Crop Sciences, Georg-August-University Göttingen, Göttingen, Germany; 3 Institute of Zoology, Faculty of Biology, University of Belgrade, Belgrade, Serbia; Institut Sophia Agrobiotech, FRANCE

## Abstract

Aphids are major pests of cereal crops and a suite of hymenopteran primary parasitoids play an important role in regulating their populations. However, hyperparasitoids may disrupt the biocontrol services provided by primary parasitoids. As such, understanding cereal aphid-primary parasitoid-hyperparasitoid interactions is vital for a reliable parasitoid-based control of cereal aphids. For this, the ability to identify the different primary and hyperparasitoid species is necessary. Unfortunately, this is often difficult due to a lack of morphologically diagnostic features. DNA sequence-based species identification of parasitoids can overcome these hurdles. However, comprehensive DNA sequence information is lacking for many of these groups, particularly for hyperparasitoids. Here we evaluate three genes [cytochrome *c* oxidase subunit I (COI), 16S ribosomal RNA (16S) and 18S ribosomal RNA (18S)] for their suitability to identify 24 species of primary parasitoids and 16 species of hyperparasitoids associated with European cereal aphids. To identify aphelinid primary parasitoid species and hyperparasitoids, we found 16S to be more suitable compared to COI sequences. In contrast, the Aphidiinae are best identified using COI due to better species-level resolution and a more comprehensive DNA sequence database compared to 16S. The 18S gene was better suited for group-specific identification of parasitoids, but did not provide resolution at the species level. Our results provide a DNA sequence database for cereal aphid primary parasitoids and their associated hyperparasitoids in Central Europe, which will allow further improvement of our understanding of cereal aphid-primary parasitoid-hyperparasitoid interactions in relation to aphid biological control.

## Introduction

Hymenopteran endoparasitoids play an important role in biological control programmes targeting aphids and other pests in both field and greenhouse crops [1]. Cereal aphids, namely the English grain aphid *Sitobion avenae*, the bird cherry-oat aphid *Rhopalosiphum padi*, and rose-grain aphid *Metopolophium dirhodum*, have been one of the most important pests in cereal production areas over the last 30 years in Europe and elsewhere [2]. These three aphid species are attacked by a suite of natural enemies including ground and vegetation dwelling predators and various groups of hymenopteran primary parasitoids [3]. Most species within these parasitic wasps belong to the braconid subfamily Aphidiinae (Hymenoptera: Braconidae). In addition, several species within the Aphelinidae in the genus *Aphelinus* are known to attack cereal aphids [1]. It has been shown that the top-down control of these natural enemy complexes can substantially decrease densities of aphids in cereals and that primary parasitoids, along with flying predators, can exert the strongest biocontrol among animal natural enemies [4,5]. However, cereal aphid primary parasitoids are frequently attacked by hyperparasitoids [6–9], including the more specialized “true hyperparasitoids” that attack living parasitized aphids and the “mummy parasitoids” which usually parasitize the aphids after mummification [10,11]. The primary parasitoid mortality caused by hyperparasitoids decreases the efficacy of the biocontrol services exerted by aphid primary parasitoids, sometimes significantly [6,12–14]. Therefore, hyperparasitoids can play an important role in shaping the levels of aphid primary parasitoid biological control.

Accurate morphological identification of parasitoid species has been a major difficulty in biological control and community ecology studies [15]. Aphid parasitoids are small insects with a body length of typically 2–3 mm, leading to a very restricted number of morphological characters available for reliable species identification [16]. Although there are taxonomic identification keys based on morphological characters parasitoid adults, they are difficult to use and morphological identification remains problematic for non-specialist [10,17–19]. This is especially true for hyperparasitoids which belong to taxonomically diverse and highly speciose groups, whose identification is even more problematic due to their small size and reduced wing venation patterns [20]. Together with common cases of cryptic speciation, this leads to considerable problems in morphological identification and an underestimation of the species richness of hyperparasitoids and their species-specific role in ecosystems [21]. Additionally, the identification of immature parasitoids in hosts is difficult and often impossible due to a lack of morphologically-distinguishing characteristics of the egg and larval stages [15,22,23]. DNA sequence-based identification can overcome these difficulties and does not require specialized taxonomic expertise [24,25]. Molecular approaches are unaffected by delayed parasitoid emergence, host and parasitoid mortality, and can be applied for each developmental stage [25–27]. Moreover, they allow for species-specific examination of trophic interactions between primary parasitoids (e.g., multiparasitism) and between primary and secondary parasitoids (e.g., hyperparasitism)–often in a single reaction [8,15,28,29]. In aphid-parasitoid systems, recently established molecular approaches for generating diagnostic DNA sequence regions of primary and secondary parasitoids [30,31] provide a strong basis for a DNA-barcoding approach for primary and secondary parasitoid species identification in their aphid hosts. The most widely used barcoding gene in animals is the 5’ end of the cytochrome *c* oxidase subunit I gene (COI) [32], which has proven effective for the identification of taxa where morphological identification is difficult or impossible [24]. Although an extensive number of COI DNA sequences for members of the Aphidiinae have recently been generated [33], COI sequences are still unavailable for several aphidiid and aphelinid primary parasitoids of cereal aphids, as well as their associated hyperparasitoid species. In contrast, Derocles et al. [30] have provided a comprehensive molecular barcoding approach for Aphidiinae species based on the 16S ribosomal RNA gene (16S). However, there is a lack of DNA sequence information for primary parasitoids within the genus *Aphelinus* and for hyperparasitoids in general [34]. Likewise, there is only limited sequence information from the 18S ribosomal RNA (18S) gene for cereal aphid primary parasitoids and their hyperparasitoids. The 18S rRNA gene is generally more suitable for identification at higher taxonomic levels [35–37] and thus might be of interest for designing group-specific molecular markers. A comprehensive database of COI, 16S, and 18S sequences for cereal aphid parasitoids, especially *Aphelinus* species and hyperparasitoids is urgently needed.

In this study we generated DNA sequences for COI, 16S and 18S genes of hyperparasitoids, as well as additional aphelinid and aphidiid parasitoids of cereal aphids that have not been covered in previous research [30]. Newly-generated sequence data and publicly-available sequence data for these species were used to evaluate the suitability of the COI, 16S and 18S genes for the development of molecular markers to identify parasitoid and hyperparasitoid species associated with cereal aphids. Additionally, the intra- and interspecific variability of these molecular markers was assessed for both primary and hyperparasitoids across different insect groups [38], which has never been assessed so far.

## Materials and methods

### Collection of parasitoids for analysis

The aphid and parasitoid samples in present study were collected from the locations, where no specific permissions were required. The samples did not involve endangered or protected species. Adult specimens of 43 species of cereal aphid primary parasitoids and their hyperparasitoids were collected from different locations in Europe (Table 1; specimen information/providers and taxonomic authorities are provided in S1 and S2 Tables). In addition, three species of primary parasitoids (*Aphelinus mali*, *Aphidius microlophii* and *Monoctonus crepidis*) that do not parasitise cereal aphids, but can be found in cereal fields due to non-cereal aphid hosts occurring on uncultivated plants within or around cereal crops, were also collected and included. Two cereal aphid parasitoid species, *Aphelinus abdominalis* and *Aphidius colemani*, which were not found in the field, were purchased from biocontrol production facilities (Sautter & Stepper GmbH Ammerbuch, Germany and Katz Biotech AG Baruth, Germany, respectively). Similarly, DNA extracts of *Toxares deltiger* and *Praon necans* were obtained from a previous study (provided by Prof. Zeljko Tomanovic, University of Belgrade, Serbia). The parasitoid adults were individually stored in 98% ethanol and morphologically identified by specialists using multiple taxonomic keys [17,39–52]. In total, 5 species of Aphelinidae, 23 species Aphidiidae, and 16 species of hyperparasitoids (belonging to several families) were obtained for analysis (see S1 and S2 Tables).

**Table 1:** Aphelinidae, Aphidiinae and hyperparasitoid species considered in this study. For each species the number of COI, 16S and 18S sequences generated from adult parasitoids is provided. Non-cereal aphid parasitoids are marked with *; parasitoid species which attack cereal aphids on their winter host plant are marked with **.

Organism group	Family/Subfamily	Species	COI sequences		16S sequences		18S sequences
			~658 bp	~288 bp	~468 bp	~342 bp	~1059 bp
Primary parasitoids	Aphelinidae	*Aphelinus abdominalis*	5		4		2
		*Aphelinus asychis*	1		1		1
		*Aphelinus chaonia*	3		3		3
		Aphelinus mali*	3		2		1
		*Aphelinus varipes*	2		1		1
	Aphidiinae	*Adialytus ambiguus*	1			2	2
		*Aphidius avenae*	5				4
		*Aphidius colemani*	2				2
		*Aphidius ervi*	5			2	3
		*Aphidius matricariae*	5		2		3
		Aphidius microlophii*	4				3
		*Aphidius rhopalosiphi*	9		1	2	4
		*Aphidius uzbekistanicus*	2				2
		*Binodoxys angelicae*	2				
		Diaeretiella rapae**	10				3
		Ephedrus persicae**	3			2	2
		*Ephedrus plagiator*	5				4
		*Lipolexis gracilis*	1				2
		*Lysiphlebus fabarum*	5				3
		*Lysiphlebus testaceipes*	5			2	2
		Monoctonus crepidis*	3				2
		*Praon abjectum*	2			2	1
		*Praon gallicum*	4				2
		*Praon necans*	2			2	4
		*Praon volucre*	4				2
		*Toxares deltiger*			1		1
		Trioxys auctus**	1		1		2
		*Trioxys* sp. A	2				1
Hyperparasitoids	Encyrtidae	*Syrphophagus aphidivorus*		5			2
	Figitidae	*Alloxysta brachyptera*					
		*Alloxysta brevis*	2		1		2
		*Alloxysta fulviceps*	3			3	2
		*Alloxysta pedestris*	1			1	1
		*Alloxysta victrix*	5		3	1	4
		*Alloxysta* sp. A	1		1		1
		*Alloxysta* sp. B	1				
		*Alloxysta* sp. C				1	
		*Phaenoglyphis villosa*	5			4	3
	Megaspillidae	*Dendrocerus carpenteri*		5	1	3	2
		*Dendrocerus laticeps*		2	2		2
	Pteromalidae	*Asaphes suspensus*	1		5		3
		*Asaphes vulgaris*	3		4		3
		*Coruna clavata*			3		2
		*Pachyneuron aphidis*	4		3		4
		*Pachyneuron formosum*		1	1		
		*Pachyneuron muscarum*	1		3		2
		*Pachyneuron solitarium*	1		2		3

### DNA extraction, PCR and sequencing

To keep the morphological features of the individuals intact, all parasitoids were non-destructively incubated in a solution made up of 180 μl ATL buffer (Qiagen, Hilden, Germany) and 20 μl proteinase K (20 mgml^-1^, AppliChem, Darmstadt, Germany) at 56°C for 2 h. Thereafter, the parasitoid was removed from the buffer-proteinase K solution, and DNA was extracted from the solution using the DNeasy Blood & Tissue Kit (Qiagen) following the manufacturer’s instructions. Within every batch of 48 samples, two extraction negative controls were included to check for potential DNA cross-contamination. These negative DNA extraction controls were tested using universal PCR primers using the conditions described below. All of these controls were negative.

A ~708 bp fragment of the COI gene was amplified and sequenced from the parasitoid DNA the using the universal invertebrate primers LCO-1490 and HCO-2198 [53]. Specimens which could not be amplified with these primers (mostly specimens which were stored dry for several years before being transferred into ethanol) were subjected to a PCR and sequenced using the general invertebrate forward primer C1-J-1859 [54] and the reverse primer HCO-2198 [53], yielding a ~339 bp fragment. Due to the short length of these ~339 bp amplicons (compared with the commonly used ~708 bp amplicons), these short sequences were submitted to GenBank but were not used for the analysis in this study. From the 16S rDNA, a ~381 bp fragment [30] was generated using the primer LR-N-13398 version ‘5’-CGCCGTTTTATCAAAAACATGT-3” [55], and LR-J-13017 [56]. Since we could not amplify this fragment of every parasitoid species in this study, also another ~510 bp 16S fragment was amplified and sequenced using the general primers LR-N13398 version ‘5’-CACCTGTTTATCAAAAACAT-3” [54] and LR-J12888 version ‘5’-TCGATTTGAACTCARATCATGTA-3” [57], respectively. From the nuclear 18S-rRNA gene a ~1100 bp amplicon was sequenced using the general primers 18SL0001 and 18SR1100 [58]. Each 10 μl PCR contained 1.5 μl DNA extract, 5 μl 2× Multiplex PCR Master Mix (Qiagen), 1 μM of each of the respective primers and PCR-grade water to adjust the volume. The PCRs were carried out in a Master Cycler Gradient (Eppendorf, Hamburg, Germany) at 95°C for 15 min, followed by 35 cycles of 94°C for 30 s, 50°C for 90 s, 72°C for 60 s, and a final extension of 72°C for 10 min. PCR products were stained with 1 × GelRed^™^ (Biotium, Hayward, USA) and visualized on 1.5% agarose gels, purified with ExoSap-IT (Amersham Biosciences, Glattbrugg, Switzerland) following the manufacturer’s instructions, and sent to Eurofins MWG Operon (Ebersberg, Germany) for bidirectional sequencing. The DNA sequences were assembled, checked and edited using BioEdit sequence alignment editor 7.0.0 [59] and aligned using MUSCLE in MEGA6 [60]. COI sequences were aligned as codons to detect frameshift mutation and premature stop codons, which may indicate pseudogenes. COI sequences for *Aphidius matricariae* and *Binodoxys angelicae* were provide by Prof. Zeljko Tomanovic, and all publicly-available DNA sequences for the parasitoid species and genes of interest on Genbank were aligned with the sequences generated in the present study.

### Data and distance analyses

As primary parasitoids of the genus *Aphelinus* are in the same superfamily (Hymenoptera: Chalcidoidea) as hyperparasitoids of the genera *Asaphes*, *Pachyneuron*, *Coruna* and *Syrphophagus*, the evaluation and analysis of the three genes was conducted for two separate groups: 1. Aphidiinae and 2. non-Aphidiinae, the latter comprising the hyperparasitoids and the aphelinid primary parasitoids of the genus *Aphelinus*.

As some hyperparasitoid and aphelinid species are not frequently sampled and many of them have never been studied by molecular analysis, only one or two specimens and DNA sequences were obtained. Additionally, the number of available 16S and 18S DNA sequences for these infrequently sampled species was significantly lower than for the COI gene. Therefore, the suitability of the COI, 16S and 18S sequences as molecular markers for identifying parasitoid species within hyperparasitoids and aphelinids was assessed using the following approach: DNA sequence distances were calculated using a K2P distance model in MEGA6 [60]. First, the species-pairs with identical DNA sequences were listed. Second, maximum within species distance (Max-WSD) versus minimum between species distance (Min-BSD) of each gene for each species pair was plotted [38]. The species-pairs without a conservative “barcoding gap”, i.e., those species where the Max-WSD was higher than the Min-BSD, were counted as difficult to be identified using the DNA sequence (*c*.*f*. Hebert et al. [61]; Hebert et al. [32]; Valentini et al. [24]). Such species-pairs are very difficult or impossible to be correctly identified via their DNA sequences. Those species, for which only one DNA sequence was obtained, were excluded from the analysis because no within species distances could be calculated.

Additionally, overall Max-WSD of each gene within each of the two groups was calculated. The species-pairs, exhibiting a smaller Min-BSD than the overall Max-WSD, were counted and listed. When the overall Max-WSD was used as a conservative cut-off point to identify a species within its respective group, these species-pairs are more likely to be misidentified via the DNA sequencing approach. Furthermore, neighbour joining trees of each group based on the K2P distances of COI and 16S gene were generated using MEGA6 [60] with 2,000 bootstraps.

## Results

### DNA extraction, PCR and sequencing

In total, 14, 26, and 80 ~658 bp COI sequences of five aphelinid, ten hyperparasitoid and 21 aphidiid species were obtained, respectively. For *Dendrocerus carpenteri*, *Dendrocerus laticeps*, *Pachyneuron formosum* and *Syrphophagus aphidivorus* only ~288 bp COI sequences could be generated. For 16S, 11 and 39 DNA sequences of five aphelinid and 14 hyperparasitoid species, respectively, were obtained. As a comprehensive 16S sequence database for the aphidiids has been generated previously [30], only a few additional DNA sequences were generated in the present study (i.e. 19 sequences for 10 species). For 18S, 53, eight and 35 DNA sequences were obtained from 21 aphidiid, five aphelinid and 14 hyperparasitoid species, respectively (Table 1; GenBank accession number see S2 Table).

For evaluation of the COI gene as a molecular marker, only the ~658 bp DNA sequences were used, as the shorter fragments do not provide enough diagnostic characters for analysis. For 16S, all 468 bp and 342 bp DNA sequences were aligned and the 342 bp long region resulting from the overlap of these two fragments was used for further analyses. To supplement the newly generated DNA sequences, 124, 71 and 3 sequences of COI, 16S and 18S were retrieved from GenBank, respectively (Table 2; GenBank accession number see S3 Table). Of all the COI sequences used in our analyses, 59.7% (40 DNA sequences) and 45.2% (80 DNA sequences) from non-Aphidiinae (*Aphelinus*/hyperparasitoid) and Aphidiinae species, respectively, were generated during this study. For 16S, 94.3% (50 DNA sequences) and 22.1% (19 DNA sequences) of the non-Aphidiinae and Aphidiinae DNA sequences were generated here. Similarly, for 18S, 97.7% (43 DNA sequences; non-Aphidiinae) and 96.4% (53 DNA sequences; Aphidiinae) were generated in the present study (Table 2).

**Table 2:** Aphelinidae, Aphidiinae and hyperparasitoid species considered in this study. For each species the number of COI, 16S and 18S sequences generated from adult parasitoids and retrieved from GenBank is provided. Non-cereal aphid parasitoids are marked with *; parasitoid species which attack cereal aphids on their winter plant host are marked with **.

Organism group	Family/Subfamily	Species	COI sequneces		16Ssequences		18S sequences	
			From specimens	From GenBank	From specimens	From GenBank	From specimens	From GenBank
Primary Parasitoid	Aphelinidae	*Aphelinus abdominalis*	5	2	4		2	
		*Aphelinus asychis*	1		1	2	1	
		*Aphelinus chaonia*	3		3		3	
		Aphelinus mali*	3		2		1	
		*Aphelinus varipes*	2	4	1	2	1	
	Aphidiinae	*Adialytus ambiguous*	1	9	2		2	
		*Aphidius avenae*	5	2		2	4	
		*Aphidius colemani*	2	7		3	2	
		*Aphidius ervi*	5	8	2	5	3	
		*Aphidius matricariae*	5	1	2	3	3	
		Aphidius microlophii*	4	3		2	3	
		*Aphidius rhopalosiphi*	9	12	3	2	4	1
		*Aphidius uzbekistanicus*	2	3		2	2	
		*Binodoxys angelicae*	2	2		2		
		Diaeretiella rapae**	10	4		9	3	
		Ephedrus persicae**	3		2	1	2	
		*Ephedrus plagiator*	5	4		3	4	
		*Lipolexis gracilis*	1	2		3	2	
		*Lysiphlebus fabarum*	5	23		9	3	
		*Lysiphlebus testaceipes*	5	5	2	6	2	1
		Monoctonus crepidis*	3	3		2	2	
		*Praon abjectum*	2	3	2		1	
		*Praon gallicum*	4	1		2	2	
		*Praon necans*	2		2	1	4	
		*Praon volucre*	4	4		10	2	
		*Toxares deltiger*		1	1		1	
		Trioxys auctus**	1		1		2	
		*Trioxys* sp. A	2				1	
Hyperparasiotid	Encyrtidae	*Syrphophagus aphidivorus*		3			2	
	Figitidae	*Alloxysta brachyptera*		1				
		*Alloxysta brevis*	2		1		2	
		*Alloxysta fulviceps*	3	3	2		2	
		*Alloxysta pedestris*	1	1	1		1	
		*Alloxysta victrix*	5	1	4		4	
		*Alloxysta* sp. A	1		1		1	
		*Alloxysta* sp. B	1					
		*Alloxysta* sp. C			1			
		*Phaenoglyphis villosa*	5	1	4		3	
	Megaspillidae	*Dendrocerus carpenteri*		3	4		2	
		*Dendrocerus laticeps*			2		2	
	Pteromalidae	*Asaphes suspensus*	1	1	5		3	1
		*Asaphes vulgaris*	3	4	4		3	
		*Coruna clavata*		1	3		2	
		*Pachyneuron aphidis*	4	2	3		4	
		*Pachyneuron formosum*			1			
		*Pachyneuron muscarum*	1		3		2	
		*Pachyneuron solitarium*	1		2		3	

### Distance analyses

The overall within species K2P distances of COI, 16S and 18S in the non-Aphidiinae were 0.00–0.17 (0.03_mean_ ± 0.004_se_), 0.00–0.02 (0.003_mean_ ± 0.0008_se_) and 0.00–0.01 (0.001_mean_ ± 0.0005_se_), respectively, whereas in the Aphidiinae they were 0.00–0.08 (0.006_mean_ ± 0.0002_se_), 0.00–0.02 (0.003_mean_ ± 0.0004_se_) and 0.00, respectively. The between species distances of COI, 16S and 18S in the non-Aphidiinae were 0.02–0.42 (0.22_mean_ ± 0.001_se_), 0.01–0.40 (0.19_mean_ ± 0.002_se_), and 0.00–0.08 (0.03_mean_ ± 0.0005_se_), respectively, and for species of the Aphidiinae group 0.00–0.26 (0.13_mean_ ± 0.0004_se_), 0.00–0.15 (0.08_mean_ ± 0.0006_se_), and 0.00–0.06 (0.03_mean_ ± 0.0005_se_), respectively.

In the non-Aphidiinae, the COI and 16S sequences were different for all species investigated. In contrast, five species groups, including 13 species, showed no difference in their 18S rDNA sequences within species groups (Table 3). In the Aphidiinae, two groups of species, *Aphidius ervi/Aphidius microlophii* and *Praon abjectum/Praon volucre*, had identical COI sequences within each group. For 16S, the same was true for the groups, *A*. *ervi/A*. *microlophii/Aphidius rhopalosiphi* and *P*. *abjectum/P*. *volucre*, while for 18S five groups, comprising 15 species, showed the same sequences for this gene within each of the group (Table 3).

**Table 3:** Parasitoid species-groups that have identical sequences in COI, 16S and 18S within Aphidiinae and non-Aphidiinae (*Aphelinus*/hyperparasitoids) parasitoids. Non-cereal aphid parasitoids are marked with *; parasitoid species which attack cereal aphids on their winter plant host are marked with **.

Gene	Group	Species-group
COI	Aphidiinae	*Aphidius ervi*, Aphidius microlophii*
		*Praon abjectum*, *Praon volucre*
16S	Aphidiinae	*Aphidius ervi*, Aphidius microlophii*, *Aphidius rhopalosiphi*
		*P*. *abjectum*, *P*. *volucre*
18S	Aphidiinae	*Adialytus ambiguus*, *Lysiphlebus fabarum*, *Lysiphlebus testaceipes*
		*Aphidius ervi*, Aphidius microlophii*, *Aphidius rhopalosiphi*, *D*. rapae**
		*Aphidius matricariae*, *A*. *rhopalosiphi*, *Aphidius uzbekistanicus*, *D*. rapae**
		*Trioxys* sp. A, Trioxys auctus**
		*P*. *abjectum*, *Praon gallicum*, *Praon necans*, *P*. *volucre*
	non-Aphidiinae	*Aphelinus abdominalis*, *Aphelinus asychis*, *Aphelinus chaonia*, Aphelinus mali*, *Aphelinus varipes*
		*Asaphes suspensus*, *Asaphes vulgaris*
		*Coruna clavata*, *Pachyneuron aphidis*
		*Dendrocerus carpenteri*, *Dendrocerus laticeps*
		*Pachyneuron muscarum*, *Pachyneuron solitarium*

**Fig 1:**
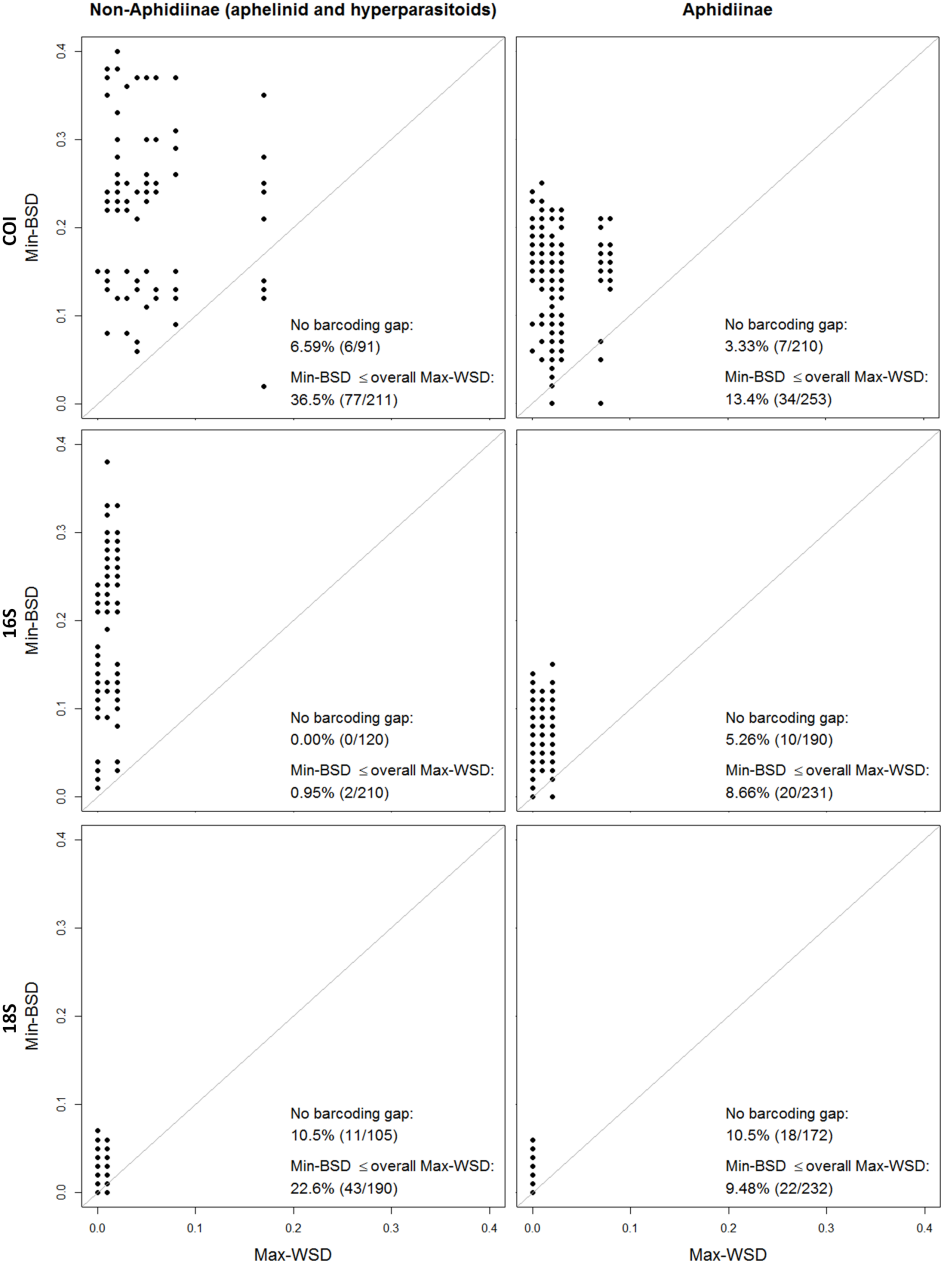
Comparison of maximum within species distance (Max-WSD; X-axis) and minimum between species distance (Min-BSD, Y-axis) of non-Aphidiinae (aphelinid/hyperparasitoid) and Aphidiinae parasitoids for COI, 16S and 18S gene sequences. The percentages of species-pairs which have a Min-BSD smaller than the Max-WSD (“No barcoding gap”) and a Min-BSD smaller than the overall Max-WSD (“Min-BSD ≤ overall Max-WSD”) are shown. Points above the diagonal represent cases where the species pairs have barcoding gap.

**Fig 2:**
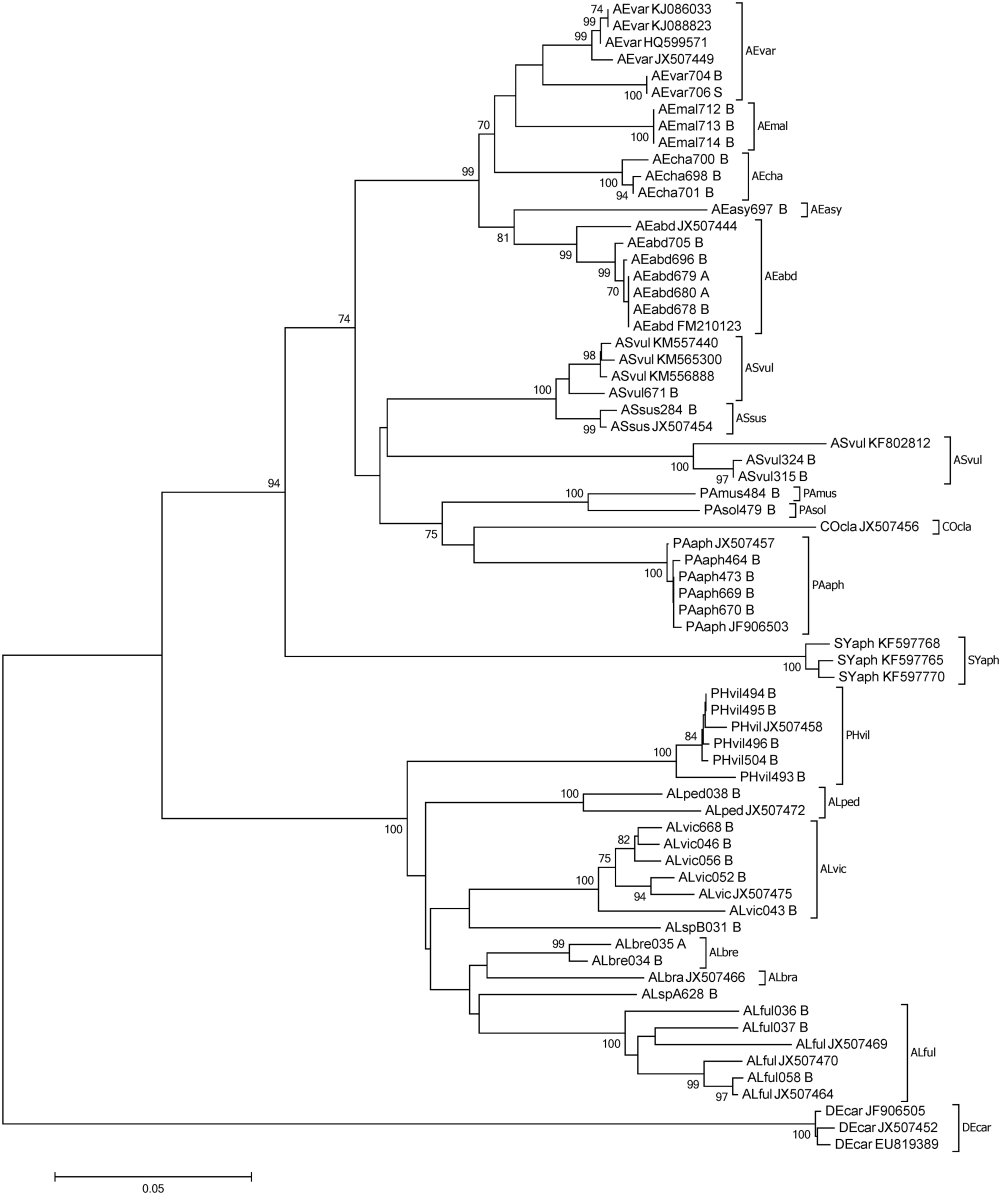
Neighbour joining tree of the non-Aphidiinae parasitoids based on sequences of the 5’-region of the cytochrome *c* oxidase I gene (COI). Bootstrap values (≥ 70%) are indicated on branches. Species abbreviations: *Alloxysta brachyptera* (ALbra), *Alloxysta brevis* (ALbre), *Alloxysta fulviceps* (ALful), *Alloxysta pedestris* (ALped), *Alloxysta victrix* (ALvic), *Alloxysta* sp.A (ALspA), *Alloxysta* sp.B (ALspB), *Alloxysta* sp.C (ALspC), *Aphelinus abdominalis* (AEabd), *Aphelinus asychis* (AEasy), *Aphelinus chaonia* (AEcha), *Aphelinus mali* (AEmal), *Aphelinus varipes* (AEvar), *Asaphes vulgaris* (ASvul), *Asaphes suspensus* (ASsus), *Coruna clavata* (COcla), *Dendrocerus carpenteri* (DEcar), *Dendrocerus laticeps* (DElat), *Pachyneuron aphidis* (PAaph), *Pachyneuron formosum* (PAfor), *Pachyneuron muscarum* (PAmus), *Pachyneuron solitarium* (PAsol), *Phaenoglyphis villosa* (PHvil) and *Syrphophagus aphidivorus* (SYaph). Last letter in the specimen code indicates whether sequencing was done in both directions (“B”) or just one direction (“S”—sense strand, “A” antisense stand).

**Fig 3:**
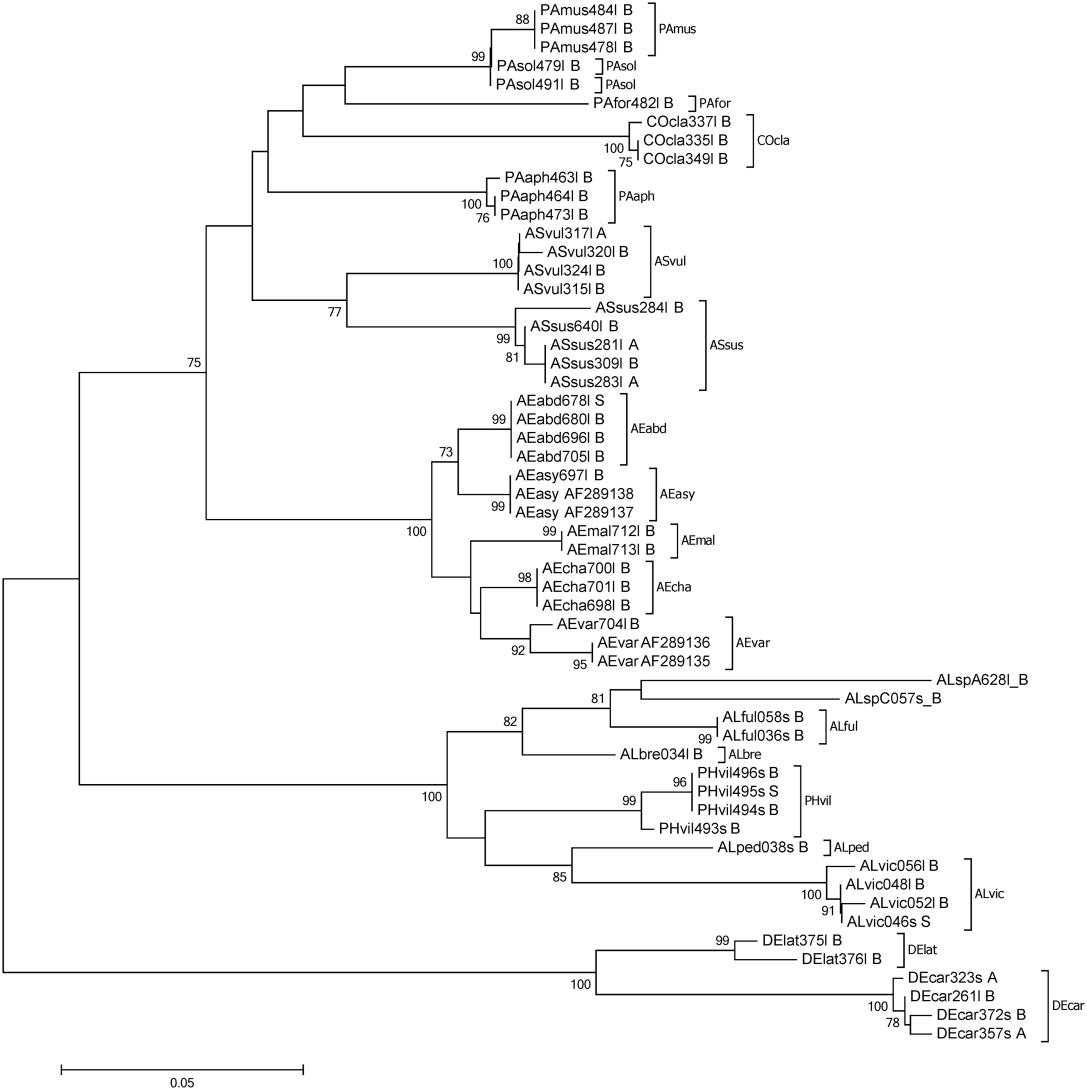
Neighbour joining tree of the non-Aphidiinae parasitoids based on sequences of the 16S ribosomal RNA gene. Bootstrap values (≥ 70%) are indicated on branches. Species abbreviations see Fig 2.

**Fig 4:**
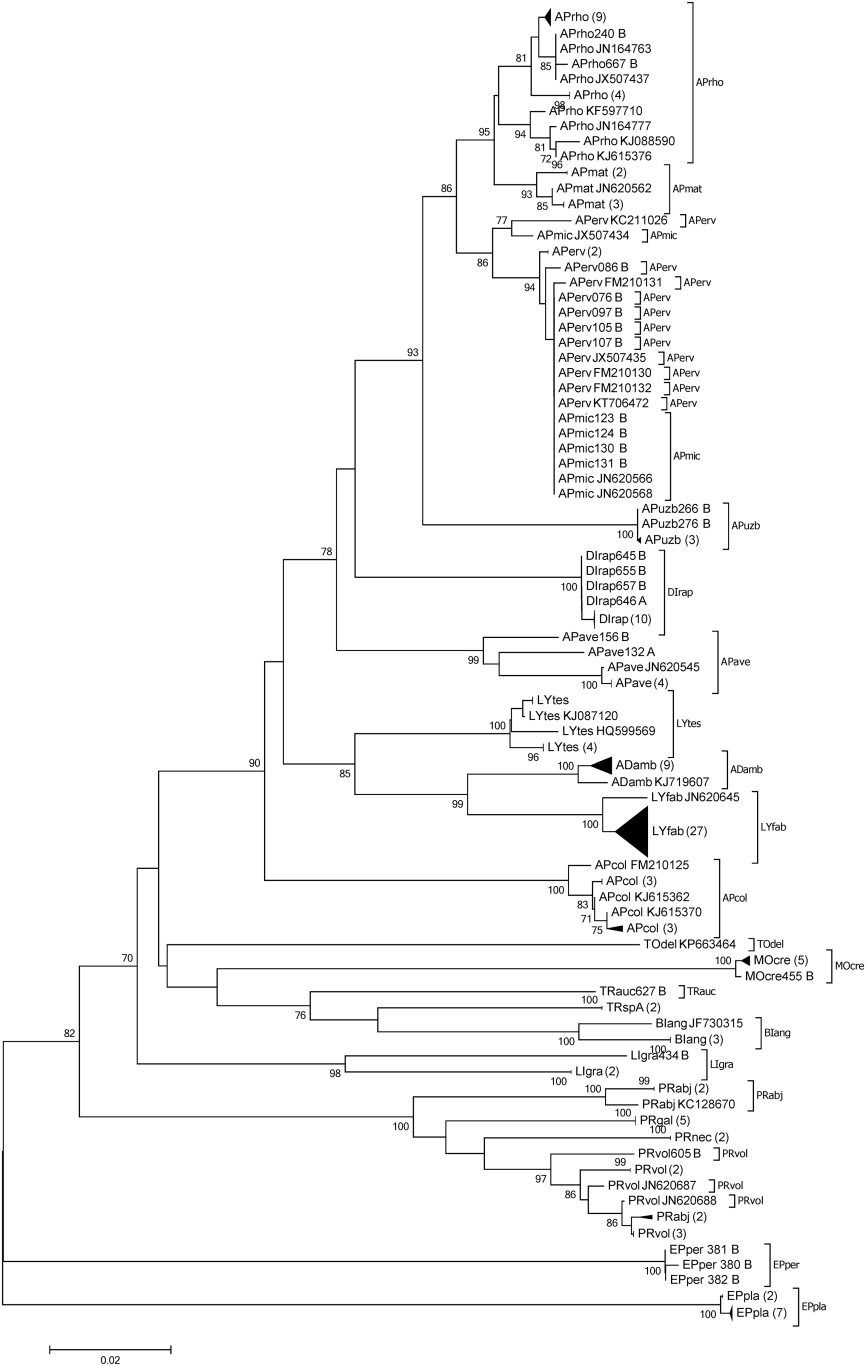
Neighbour joining tree of parasitoid species within the Aphidiinae based on sequences of the 5’-region of the cytochrome *c* oxidase I gene (COI). Bootstrap values (≥ 70%) are indicated on branches. Identical sequences and subtrees with all bootstrap values less than 70% within one species were clustered. Numbers in parentheses refer to the number of sequences include in each cluster. Species abbreviations: *Adialytus ambiguus* (ADamb), *Aphidius avenae* (APave), *Aphidius colemani* (APcol), *Aphidius ervi* (APerv), *Aphidius matricariae* (APmat), *Aphidius microlophii* (APmic), *Aphidius rhopalosiphi* (APrho), *Aphidius uzbekistanicus* (APuzb), *Binodoxys angelicae* (BIang), *Diaeretiella rapae* (DIrap), *Ephedrus persicae* (EPper) *Ephedrus plagiator* (EPpla), *Lipolexis gracilis* (LIgra), *Lysiphlebus fabarum* (LYfab), *Lysiphlebus testaceipes* (LYtes), *Monoctonus crepidis* (MOcre), *Praon abjectum* (PRabj), *Praon gallicum* (PRgal), *Praon necans* (PRnec), *Praon volucre* (PRvol), *Toxares deltiger* (TOdel), *Trioxys auctus* (TRaus) and *Trioxys* sp. A (TRspA). Last letter in the specimen code indicates whether sequencing was done in both directions (“B”) or just one direction (“S”—sense strand, “A”—antisense stand).

**Fig 5:**
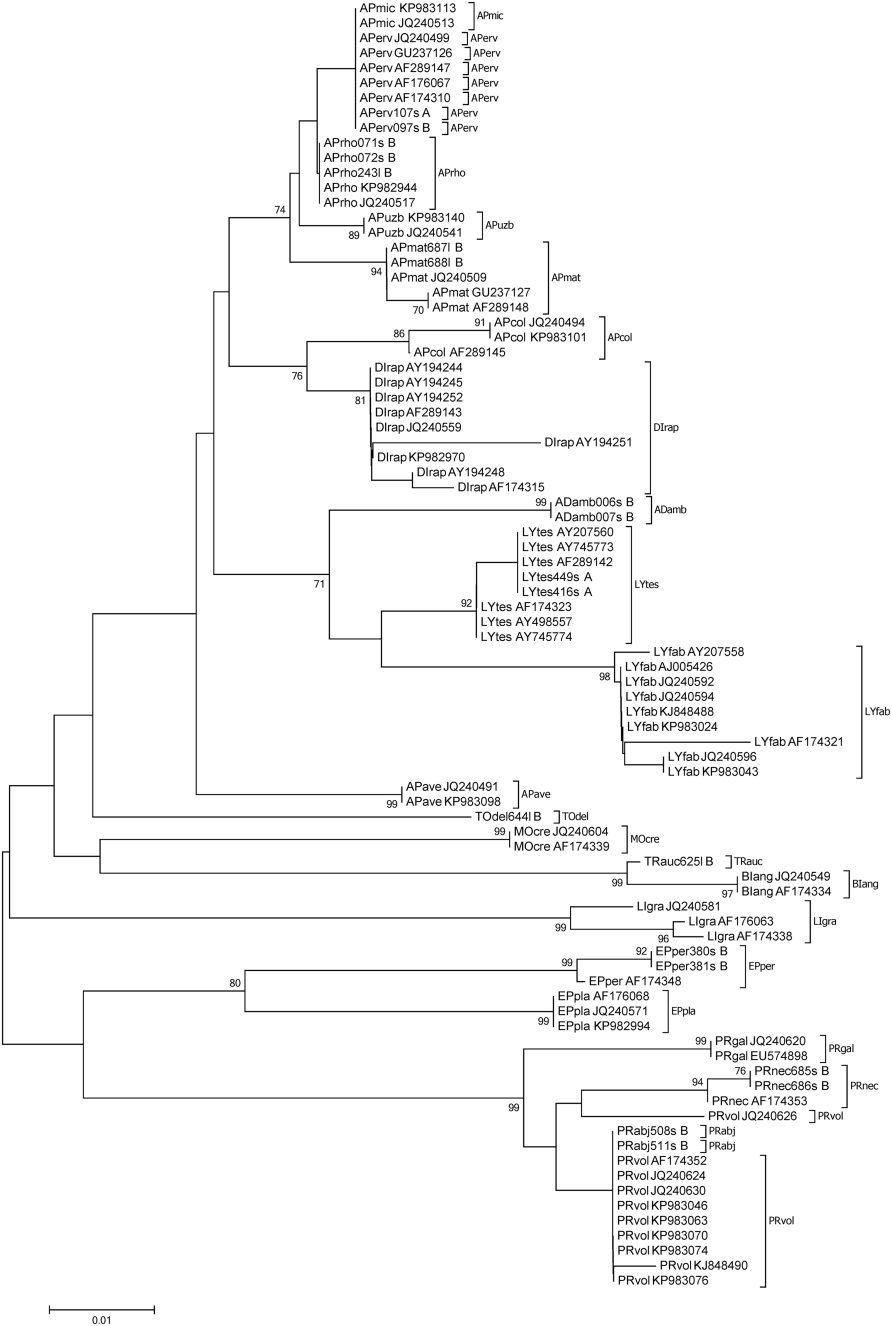
Neighbour joining tree of parasitoid species within the Aphidiinae based on 16S ribosomal RNA gene sequences. Bootstrap values (≥ 70%) are indicated on branches. Species abbreviations see Fig 4.

A barcoding gap between all species within the non-Aphidiinae group was found for 16S (n = 120), whereas there were species pairs lacking such a gap for COI and 18S (6.59%, n = 91 and 10.5%, n = 105, respectively). Within the Aphidiinae, compared to COI (3.33%, n = 210), a higher percentage of species-pairs had no barcoding gap on 16S (5.26%, n = 190). For 18S the species pairs which showed no barcoding gap was 10.5% (n = 172) (Fig 1). Species-pairs with a Min-BSD smaller than the overall Max-WSD within the non-Aphidiinae was 0.95% (n = 210) for 16S, compared to 36.5% (n = 211) and 22.6% (n = 190) for COI and 18S, respectively. Within the Aphidiinae this relationship was found for 8.66% (n = 231), 13.4% (n = 253), and 9.48% (n = 232) for 16S, COI, and 18S, respectively (Fig 1). In the neighbour joining tree, the COI and 16S sequences of non-Aphidiinae group clustered according to the morphologically assigned species. However, for COI the clade of *Asaphes vulgaris*, which had an average within species distance of 0.09, was not differentiated from *Asaphes suspensus* (Figs 2 and 3). For the Aphidiinae group, *A*. *ervi/A*. *microlophii* and *P*. *abjectum/P*. *volucre* were in the same clades, respectively, for both the COI and 16S sequences (Figs 4 and 5).

## Discussion

The present study provides new possibilities for species identification in cereal aphid primary parasitoids and their hyperparasitoids in Central Europe. We significantly increased the publicly-available 16S and 18S sequence information, especially for species of the genus *Aphelinus* and for hyperparasitoids associated with aphid primary parasitoids. Moreover, the COI sequence information for aphidiid and non-aphidiid parasitoid taxa has been significantly expanded by the sequence data generated in the present study. Altogether, a DNA sequence database for COI, 16S and 18S of 24 and 16 cereal aphid primary parasitoid and hyperparasitoid species, respectively, is now publicly available (Genbank accession numbers see S2 and S3 Tables). However, for the two hyperparasitoid species *Syrphophagus aphidivorus* and *Alloxysta brachyptera*, no 16S and 18S sequence information, respectively, could be generated. In addition, for the two hyperparasitoid species *D*. *laticeps* and *P*. *formosum*, only a short part (~288 bp) of the COI barcoding sequence could be obtained.

In general, the COI and 16S sequence fragments are more suitable for species identification compared with the 18S sequences which were found to be most conserved among the parasitoid species investigated. This is not surprising as this gene region maintains the secondary structure of rRNA molecules and therefore a low sequence variability is expected [62,63]. Additionally, mitochondrial genes evolve faster than nuclear ones such as 18S [54,62]. Due to its reduced interspecific sequence variability the 18S gene is less suitable for species-specific detection and identification of parasitoids, but may represent an interesting target for group specific detection and identification, as well as phylogeny at higher taxonomic level [62]. As there are more copies of the mitochondrial COI and 16S genes per cell compared to the nuclear 18S gene, mitochondrial DNA fragments have a higher chance to be amplified, even from degraded tissues of long-term stored insects [63]. Consequently, COI and 16S genes have been widely used for molecular detection and identification of parasitoids [31,34], However, mitochondrial genes are usually strongly associated with maternal inheritance, which can lead to an intraspecific overestimate of the distance by disequilibria selection of females and maternally inherited symbionts. On the other hand, interspecific hybridization and symbiont infections also transfer mitochondrial genes among evolutionary groups, leading an underestimate of interspecific distance [64]. Nevertheless, mitochondrial genes are suitable targets for species detection and identification [32].

From some rarely sampled species only one or two sequences could be obtained. This low sample number should be taken into account when interpreting our findings as previous work has shown that a good estimation of the maximum pairwise distance in DNA barcoding should be based on ≥ 20 individuals per species [65]. Also, sample number and between species distance in DNA barcoding has been shown to be positively correlated [66]. However, the barcode gap is usually not affected by sample size and even a relatively small barcode library can be used for identification of specimens collected from larger geographic scale [66]. This suggests that the present findings are still reliable, although in some species only very few sequences could be obtained.

### Non-Aphidiinae parasitoids and hyperparasitoids

In the non-Aphidiinae, the COI standard barcoding region [67,68] was found to be more divergent within species than in the 16S sequences. Consequently, the 16S sequences were slightly better suited for species identification. This was supported primarily by sequence data from three species: *Asaphes vulgaris*, *Alloxysta fulviceps* and *Alloxysta victrix*. In *A*. *vulgaris* there was no species-pair without a barcoding gap in 16S, while the COI clades were overlapping with *Asaphes suspensus*. This was evidenced by the highly divergent COI sequences of *A*. *vulgaris* (mean within species distance 0.09). The large within species distances and the resulting neighbour joining tree of COI sequences suggest the possibility of misidentified specimens, or the existence of cryptic species. Additional studies would be necessary to clarify this situation. The second species, *A*. *fulviceps*, showed higher variability in the COI compared to 16S sequences: for example, specimens “ALful036” and “ALful058” had a COI within group distance of 0.06, whereas the 16S sequences of these two specimens were the same. Nevertheless, the mean within group distance of COI was 0.05 and 16S sequences suggested *Alloxysta* sp. A and *Alloxysta* sp. C as a possible sister group of *A*. *fulviceps*. As a COI divergence within species has been proposed to be lower than 0.022 [68], our findings indicate the possibility of cryptic speciation. Further taxonomic research is needed to confirm the status of both species. For *A*. *victrix*, the third species, the mean within group distance was 0.03 for COI, whereas the mean within group distance for 16S was only 0.006. This finding also points to the possibility of cryptic speciation within *A*. *victrix*, which is a cosmopolitan generalist. This is in accordance with findings on the patterns of antennal sensillar equipment of this species, which also suggest the existence of cryptic species in *A*. *victrix* [69]. To conclude, it is possible to use the DNA sequences of both COI and 16S to discriminate species between all non-Aphidiinae investigated here. 16S sequences perform slightly better than COI sequences for molecular identification of *Aphelinus* and hyperparasitoid species. Additionally, specimens of the primary parasitoid species, *Aphelinus varipes* were separated into two clades in the neighbour joining trees of both COI and 16S. As *A*. *varipes* has been described as a species complex [70], further taxonomic studies of both clades are suggested.

### Aphidiinae parasitoids

Overall, the COI sequences provide a slightly higher species resolution and a much larger current database [30] than 16S, suggesting that COI is better suited for species identification in Aphidiinae than 16S. On the basis of morphological differences and some biological traits such as host acceptance behaviour and host range patterns, Pennacchio and Tremblay [71] described *Aphidius microlophii* as a cryptic species within the *A*. *ervi* complex. Furthermore, Pennacchio & Temblay [71] and Pennacchio et al. [72] state that *A*. *microlophii* is a parasitoid specific to stinging nettle aphids, *Microlophium carnosum*. However, COI sequences do not separate *A*. *ervi* from *A*. *microlophii*, and 16S sequences were identical among *A*. *ervi*, *A*. *microlophii* and *A*. *rhopalosiphi*. Previous studies have also shown that *A ervi* and *A*. *microlophii* are not genetically distinct based on COI [33] and on 16S gene sequences [30]. Nevertheless, we included *A*. *microlophii* in our study, since it is still not clear if it is a separate species specific to *M*. *carnosum* on *Urtica* plants or just a specific population of *A*. *ervi* which also attacks cereal aphids.

Similarly, it was not possible to clearly differentiate between *P*. *abjectum* and *P*. *volucre* using COI and 16S sequences, although a previous study has shown that these two species are both morphologically and genetically (COI) distinct [73,74]. The high intraspecific variability in *P*. *abjectum* indicates the possibility of a species complex related to several host-associated lineages: some of our *P*. *abjectum* specimens originated from elder aphids, *Aphis sambuci*, collected from *Sambucus nigra* which is a common shrub around cereal agroecosystems. Recently, a new parasitoid species, *Praon sambuci*, which is associated with the *A*. *sambuci-S*. *nigra* system, was described [73]. Therefore, further evaluation of the taxonomic status of *P*. *abjectum*, *P*. *sambuci* and *P*. *volucre* is warranted.

The distance between two clades of *Binodoxys angelicae* on the COI gene was 0.03, also suggesting a possible species complex. Interestingly, apart from *Trioxys auctus*, which is the only known *Trioxys* cereal aphid parasitoid in Europe, a *Trioxys* sp. A clade was found. The COI distances from the two specimens on this clade to *B*. *angelicae* and *T*. *auctus* were 0.08. As *Trioxys* sp. A was reared from bird cherry-oat aphids, *Rhopalosiphum padi*, we suggest that it could be some unknown *Trioxys* cereal aphid parasitoid from Europe or some exotic species accidentally introduced to Europe. The later seems likely, *Trioxys sunnysidensis* reared from *R*. *padi* has recently been described in central Washington, USA [75]. Nevertheless, further studies are suggested to address these questions.

### DNA based identification of Aphidiinae and non-Aphidiinae associated with cereal aphids

For DNA-based identification of parasitoids within their hosts, PCR assays which employ primers amplifying the DNA of parasitoids but not of the host can be used. There are two ways of detecting and identifying these parasitoids: either using species-specific primers (e.g. Gariepy and Messing [28]; Macfadyen et al. [8]; Traugott et al. [7]) or parasitoid group-specific primers which amplify a DNA fragment which allows sequence-based discrimination of the parasitoid species [30]. For parasitoid identification using species-specific primers, the higher divergence within species of non-aphidiid parasitoids in COI gene sequences can make primer design challenging. For these species, 16S sequences would be better suited for the establishment of species-specific primers [31]. For the Aphidiinae, however, the standard barcode region COI seems to be best suited for species-specific primer design [7,8,31]. The comprehensive COI and 16S sequence information provided by the present study is an important requirement for designing species-specific primers for cereal aphid parasitoids. For the DNA sequence-based identification approach using group-specific parasitoid primers, such as Sanger sequencing and next generation sequencing (NGS), the 16S sequences are better suited than the more divergent COI sequences. The 16S sequences contain enough variability between species to allow for sequence-based identification of the cereal aphid parasitoid species considered in our study. Such 16S-based group-specific primers for non-Aphidiinae and Aphidiinae have just recently been developed [30,31], and the 16S sequences for cereal aphid primary parasitoids and their hyperparasitoids provided by this study represents an important reference DNA sequence database.

Overall, this research expands the sequence database for parasitoids and hyperparasitoids associated with cereal aphids, and provides a foundation for additional molecular studies aimed at gaining a better understanding of the biocontrol services provided by aphid parasitoids in crop and non-crop ecosystems.

## Supporting information

S1 Table Taxonomic authorities of the organisms used in this study. (DOCX)

S2 Table Parasitoid specimens used in this study. (XLSX)

S3 Table Parasitoid DNA sequences retrieved from GenBank for this study. (DOCX)
