# Supplementary material for: Evaluation of three molecular markers for identification of European primary parasitoids of cereal aphids and their hyperparasitoids
Source: PLoS One. 2017 May 31;12(5):e0177376. doi: 10.1371/journal.pone.0177376 (PMC5451020; doi:10.1371/journal.pone.0177376)
Supplement: S1 Table — (DOCX) [file pone.0177376.s001.docx]

**Table S1. Taxonomic authorities of the organisms used in this study.**

| **Organism group** | **Family** | **Species/Genus** | **Taxonomic authority** |
| --- | --- | --- | --- |
| primary parasitoid | Aphelinidae | *Aphelinus abdominalis* | Dalman 1820 |
|  |  | *Aphelinus asychis* | Walker 1839 |
|  |  | *Aphelinus chaonia* | Walker 1839 |
|  |  | *Aphelinus mali* | Haldeman 1851 |
|  |  | *Aphelinus varipes* | Förster 1841 |
|  | Braconidae | *Adialytus ambiguus* | Haliday 1834 |
|  |  | *Aphidius avenae* | Haliday 1834 |
|  |  | *Aphidius colemani* | Viereck 1912 |
|  |  | *Aphidius ervi* | Haliday 1834 |
|  |  | *Aphidius matricariae* | Haliday 1834 |
|  |  | *Aphidius microlophii* | Pennacchio & Tremblay 1987 |
|  |  | *Aphidius rhopalosiphi* | Stefani-Perez 1902 |
|  |  | *Aphidius uzbekistanicus* | Luzhetzki 1960 |
|  |  | *Binodoxys angelicae* | Haliday 1833 |
|  |  | *Diaeretiella rapae* | M'Intosh 1855 |
|  |  | *Ephedrus persicae* | Froggatt 1904 |
|  |  | *Ephedrus plagiator* | Nees 1811 |
|  |  | *Lipolexis gracilis* | Förster 1862 |
|  |  | *Lysiphlebus fabarum* | Marshall 1896 |
|  |  | *Lysiphlebus testaceipes* | Cresson 1880 |
|  |  | *Monoctonus crepidis* | Haliday 1834 |
|  |  | *Praon abjectum* | Haliday 1833 |
|  |  | *Praon gallicum* | Starý 1971 |
|  |  | *Praon necans* | Mackauer 1959 |
|  |  | *Praon volucre* | Haliday 1833 |
|  |  | *Toxares deltiger* | Haliday 1833 |
|  |  | *Trioxys auctus* | Haliday 1833 |
| Hyperparasiotid | Encyrtidae | *Syrphophagus aphidivorus* | Mayr 1876 |
|  | Figitidae | *Alloxysta brachyptera* | Hartig 1840 |
|  |  | *Alloxysta brevis* | Thomson 1862 |
|  |  | *Alloxysta fulviceps* | Curtis 1838 |
|  |  | *Alloxysta pedestris* | Curtis 1838 |
|  |  | *Alloxysta victrix* | Westwood 1833 |
|  |  | *Phaenoglyphis villosa* | Hartig 1841 |
|  | Megaspillidae | *Dendrocerus carpenteri* | Curtis 1829 |
|  |  | *Dendrocerus laticeps* | Hedicke 1929 |
|  | Pteromalidae | *Asaphes suspensus* | Nees 1834 |
|  |  | *Asaphes vulgaris* | Walker 1834 |
|  |  | *Coruna clavata* | Walker 1833 |
|  |  | *Pachyneuron aphidis* | Bouché 1834 |
|  |  | *Pachyneuron formosum* | Walker 1833 |
|  |  | *Pachyneuron muscarum* | L. 1758 |
|  |  | *Pachyneuron solitarium* | Hartig 1838 |
