# Supplementary material for: Evaluation of three molecular markers for identification of European primary parasitoids of cereal aphids and their hyperparasitoids
Source: PLoS One. 2017 May 31;12(5):e0177376. doi: 10.1371/journal.pone.0177376 (PMC5451020; doi:10.1371/journal.pone.0177376)
Supplement: S3 Table — (DOCX) [file pone.0177376.s003.docx]

**Table S3. Parasitoid DNA sequences retrieved from GenBank for this study.** Non-cereal aphid parasitoids are marked with *; parasitoid species which attack cereal aphids on their winter plant host are marked with **.

| **Gene** | **Organism group** | **Family/Subfamily** | **Species** | **GenBank accession number** |
| --- | --- | --- | --- | --- |
| COI | Primary Parasitoid | Aphelinidae | *Aphelinus abdominalis* | FM210123, JX507444 |
|  |  |  | *Aphelinus varipes* | HQ599571, JX507449, KJ086033, KJ088823 |
|  |  | Aphidiinae | *Adialytus ambiguus* | KJ719605, KJ719606, KJ719607, KJ719608, KJ719609, KJ719610, KJ719611, KJ719612, KJ719613 |
|  |  |  | *Aphidius avenae* | EU819392, EU819393, JN164785, JN620545, JN620547, JQ723406, JQ723408 |
|  |  |  | *Aphidius colemani* | FM210125, FM210126, JN620548, JN620549, KJ615362, KJ615370-KJ615373 |
|  |  |  | *Aphidius ervi* | FM210130, FM210131, FM210132, FM210134, JX507435, KC211025, KC211026, KT706472 |
|  |  |  | *Aphidius matricariae* | JN620562 |
|  |  |  | *Aphidius microlophii** | JN620566, JN620568, JX507434, |
|  |  |  | *Aphidius rhopalosiphi* | EU819401, EU819402, EU819403-EU819406, JN164753, JN164754, JN164763, JN164777, JN164778, JN620570-JN620572, JX507437, KF597710, KJ088590, KJ615376 |
|  |  |  | *Aphidius uzbekistanicus* | JN164735, JN164746, JN620594, KF597706 |
|  |  |  | *Binodoxys angelicae* | JF730315, JN620603 |
|  |  |  | *Diaeretiella rapae*** | JF730316 |
|  |  |  | *Diaeretiella rapae*** | JN620613, JN620615, KF802814 |
|  |  |  | *Ephedrus plagiator* | JN620623, JN620625, JN620627, JX507443 |
|  |  |  | *Lipolexis gracilis* | JN620635, JN620636 |
|  |  |  | *Lysiphlebus fabarum* | JF730314, JN620645, JN620647, JN620652, JQ723415, JX507442, KC237766-KC237768, KF597685, KF597686, KF597688-KF597690, KF597681, KF597692, KM408522, KP663444, KP663448-KP663450, KP663455-KP663457, KP663459 |
|  |  |  | *Lysiphlebus testaceipes* | FM210176, HQ599569, JN620653, JN620655, JX470530, JX470531, JX470533, KC237764, KC237765, KJ087120, KJ090001 |
|  |  |  | *Monoctonus crepidis** | JN620660, JN620661, JN620662 |
|  |  |  | *Praon abjectum* | KC128669- KC128671 |
|  |  |  | *Praon gallicum* | EU574906, EU819398-EU819400, JN620679 |
|  |  |  | *Praon volucre* | EU819394-EU819397, JN620687-JN620689, JN620681, KJ698487, KJ698496, KJ698504, KJ698507 |
|  |  |  | *Toxares deltiger* | EU819391, KP663464 |
|  | Hyperparasiotid | Encyrtidae | *Syrphophagus aphidivorus* | KF597765, KF597768, KF597770 |
|  |  | Figitidae | *Alloxysta brachyptera* | JX507466 |
|  |  |  | *Alloxysta fulviceps* | JX507464, JX507469, JX507470 |
|  |  |  | *Alloxysta pedestris* | JX507472 |
|  |  |  | *Alloxysta victrix* | EU819388, JX507475 |
|  |  |  | *Phaenoglyphis villosa* | JX507458 |
|  |  | Megaspillidae | *Dendrocerus carpenteri* | EU819389, JF906505, JX507452 |
|  |  | Pteromalidae | *Asaphes suspensus* | JX507454 |
|  |  |  | *Asaphes vulgaris* | EU819407, EU819408, JX507453, KF802812, KF802813, KM556888, KM557440, KM561413, KM565300 |
|  |  |  | *Coruna clavata* | JX507456 |
|  |  |  | *Pachyneuron aphidis* | JF906503, JX507457, KF597737, KF597738, KF597739, KF597740, KF597741 |
| 16S | Primary Parasitoid | Aphelinidae | *Aphelinus asychis* | AF289137, AF289138 |
|  |  |  | *Aphelinus varipes* | AF289135, AF289136 |
|  |  |  | *Aphidius avenae* | JQ240491, KP983098 |
|  |  |  | *Aphidius colemani* | AF289145, JQ240494, KP983101 |
|  |  |  | *Aphidius ervi* | AF174310, AF176067, AF289147, GU237126, JQ240499 |
|  |  |  | *Aphidius matricariae* | AF289148, GU237127, JQ240509 |
|  |  |  | *Aphidius microlophii** | JQ240513, KP983113 |
|  |  |  | *Aphidius rhopalosiphi* | JQ240517, KP982944 |
|  |  |  | *Aphidius uzbekistanicus* | JQ240541, KP983140 |
|  |  |  | *Binodoxys angelicae* | AF174334, JQ240549 |
|  |  |  | *Diaeretiella rapae*** | AF174315, AF289143, AY194244, AY194245, AY194248, AY194251, AY194252, JQ240559, KP982970 |
|  |  |  | *Ephedrus persicae*** | AF174348 |
|  |  |  | *Ephedrus plagiator* | AF176068, JQ240571, KP982994 |
|  |  |  | *Lipolexis gracilis* | AF174338, AF176063, JQ240581 |
|  |  |  | *Lysiphlebus fabarum* | AF174321, AJ005426, AY207558, JQ240592, JQ240594, JQ240596, KJ848488, KP983024, KP983043 |
|  |  |  | *Lysiphlebus testaceipes* | AF174323, AF289142, AY207560, AY498557, AY745773, AY745774 |
|  |  |  | *Monoctonus crepidis** | AF174339, JQ240604 |
|  |  |  | *Praon gallicum* | EU574898, JQ240620 |
|  |  |  | *Praon necans* | AF174353 |
|  |  |  | *Praon volucre* | AF174352, JQ240624, JQ240626, JQ240630, KJ848490, KP983046, KP983063, KP983070, KP983074, KP983076 |
| 18S | Primary Parasitoid | Aphelinidae | *Aphelinus asychis* | JN623060 |
|  |  | Aphidiinae | *Adialytus ambiguus* | AJ009317 |
|  |  |  | *Aphidius colemani* | AJ009318 |
|  |  |  | *Aphidius ervi* | AJ009321 |
|  |  |  | *Aphidius matricariae* | AJ009324 |
|  |  |  | *Aphidius rhopalosiphi* | KT204373 |
|  |  |  | *Binodoxys angelicae* | AJ009349 |
|  |  |  | *Diaeretiella rapae*** | AJ009323 |
|  |  |  | *Ephedrus persicae*** | AJ009329 |
|  |  |  | *Lipolexis gracilis* | AJ009334 |
|  |  |  | *Lysiphlebus fabarum* | AJ009332 |
|  |  |  | *Lysiphlebus testaceipes* | AJ009335, AY216698 |
|  |  |  | *Praon volucre* | AJ009347 |
|  | Hyperparasiotid | Megaspillidae | *Dendrocerus carpenteri* | AY918978 |
|  |  | Pteromalidae | *Asaphes suspensus* | JN623355 |
|  |  |  | *Coruna clavata* | JN623453 |
|  |  |  | *Pachyneuron formosum* | JN623464 |
